# Dynamic prediction of the risk of recurrence in patients over 60 years of age with differentiated thyroid carcinoma

**DOI:** 10.1590/2359-3997000000146

**Published:** 2016-02-11

**Authors:** Yanina Jimena Morosán, Carina Parisi, María Agustina Urrutia, Melanie Rosmarin, Marta Schnitman, Leonardo Serrano, Wilfrido Luciani, Cristina Faingold, Fabián Pitoia, Gabriela Brenta

**Affiliations:** 1 Dr. César Milstein Hospital Buenos Aires Argentina Division of Endocrinology, Dr. César Milstein Hospital, Buenos Aires, Argentina; 2 Dr. César Milstein Hospital Buenos Aires Argentina Division of Surgery, Dr. César Milstein Hospital, Buenos Aires, Argentina; 3 Hospital de Clínicas University of Buenos Aires Buenos Aires Argentina Division of Endocrinology, Hospital de Clínicas, University of Buenos Aires, Buenos Aires, Argentina

**Keywords:** Thyroid cancer, elderly, risk re-stratification, recurrence, staging

## Abstract

**Objective:**

: The reclassification of the risk according to the response to the initial treatment makes the treatment of differentiated thyroid cancer (DTC) vary in each individual. As the influence of age on this diagnostic strategy is unknown, we have decided to assess it in adults who are over 60 years of age.

**Subjects and methods:**

: Ninety patients with DTC above 60 years old were enrolled, with total thyroidectomy plus radioiodine ablation, negative anti-thyroglobulin antibodies, follow-up ≥ 2 years and with clinical and pathological information to classify the risk of recurrence according to ATA (American Thyroid Association) and reclassify based on the response to initial therapy according to MSKCC (Memorial Sloan Kettering Cancer Center). The structural persistence at the end of the follow-up was the gold standard of our analysis.

**Results:**

: The structural persistence in ATA low, intermediate and high risk categories was 0, 38, and 100%, respectively. In the intermediate group, none of those with an excellent response to the initial treatment showed structural persistence, whereas 39% of those with an incomplete/indeterminate response showed structural persistence (p < 0.01).

**Conclusions:**

The re-stratification according to the response to the initial treatment in patients over 60 years of age with an ATA intermediate risk of recurrence allowed for the distinction of disease-free patients at the end of the follow-up from those with structural persistence and a worse clinical progression.

## INTRODUCTION

Differentiated thyroid carcinoma (DTC) generally has a favourable prognosis. Nevertheless, when DTC is detected in older adults, except in the case of the microcarcinoma, traditionally the measures to remove it are maximized, along with any possible extension it may have. Such behaviour is supported by the notion that the advanced age is a directly proportional risk factor which defines the aggressiveness of thyroid carcinomas and it is an independent prognostic factor in all thyroid cancer staging systems (1,2). Advanced age at the moment of diagnosis, together with other factors such as the presence of distant metastases, local tumour invasion, and the presence of lymph node metastases, is linked to a lower survival in thyroid cancer (3).

Age above or below 45 years has been used in the staging systems because it seems to be related to patient progression (4). Despite the fact that, regarding survival, the 7^th^ edition of the AJCC/UICC staging system allows for the accurate anticipation according to this age cut-off, it has been demonstrated that this system has a poor correlation with the recurrence of the disease (5). In addition, if we consider that older adults are those over 60 years of age (6), the current clinical practice guidelines are not sufficiently conclusive as regards the therapeutic behaviour or the follow-up of these patients (7,8).

At present there is a renewed interest in the management of thyroid cancer, where the treatment and follow-up are more targeted and individualized. With this new approach, patients with a high risk of recurrence would be more likely to benefit from an intensive treatment since most of them remain with a structural persistence or recurrence at the end of the follow-up, whereas low risk patients would receive more conservative treatments as most of them show an excellent response to the treatment (9-11). As to patients with ATA (American Thyroid Association) intermediate risk, it is much more difficult to establish which will be their progression. However, if diagnostic elements are available in the first 2 years after the thyroidectomy in order to reclassify the risk according to the response to the initial treatment, this problem tends to be solved (12). This seems to happen because many intermediate risk patients, after being re-stratified, change their status to either low or high risk which is, in general, consistent with their final progress. Although this has already been demonstrated in the general population of patients with thyroid cancer, it still has not been studied in older adult subjects who, due to the assumption that they are at a higher risk, generally undergo more intensified follow-up treatments.

For this reason, in our study we have used the risk re-stratification according to the response to the initial treatment in adults who are over 60 years of age, in order to be able to validate such system in this population of patients.

## SUBJECTS AND METHODS

### Population

Ninety patients with DTC were retrospectively included, all of them from 2 endocrinology centres, Dr. Cesar Milstein Hospital and Hospital de Clínicas, both located in the city of Buenos Aires, an iodine sufficient metropolitan area. The study period spanned from 2000 to 2013.

The only patients who were considered were those over 60 years of age who had received treatment with total thyroidectomy plus radioiodine ablation, negative anti-thyroglobulin antibodies, a follow-up period higher than or equal to 2 years, and who had the clinical and pathological information which allowed for their re-stratification.

Patients (n: 110) were excluded for the following reasons: lack of information for their appropriate follow- up, inadequate information for their initial staging, incompatible histology with differentiated thyroid carcinoma, positive anti-thyroglobulin antibodies.

Both the laboratory assessments (thyroglobulin and anti-thyroglobulin antibodies [Tg-Ab]) and the neck ultrasound scans, were performed in such institutions. All patients received treatment with TSH-suppressive dose of levothyroxine, depending on each ATA risk category.

The results of the neck ultrasound scans and the thyroglobulin and Tg-Ab assessments were recorded during the first two years of follow-up under the TSH suppressive therapy. The stimulated thyroglobulin dosing during this period was available in 88% of patients, but this was not considered as a requirement for enrolment in our study.

### Staging

All patients were staged using the 7^th^ edition of the AJCC/UICC staging system (stage I, II, III or IV) (3) and the ATA risk of recurrence (low, intermediate and high risk of recurrence) (12) (Table 1).

**Table 1:** Risk of recurrence according to ATA (12)

Low risk (all required)	Intermediate risk (any of the following)	High risk (any of the following)
Classic papillary carcinoma (including follicular variant)	Cervical lymph nodes metastases	Gross extrathyroidal extension
Confined to thyroid	Microscopic extrathyroidal extension	Tumour incomplete resection
No vascular invasion	Aggressive histology (insular, tall cell, columnar, diffuse sclerosing, follicular carcinoma)	Distant metastasis
No I131 uptake outside the thyroid bed on the post treatment scan, if done	Vascular invasion	Inappropriate increase of thyroglobulin

### Laboratory studies

The method used for measuring the thyroglobulin and Tg-Ab was chemiluminescence (Centaur and Immulite 1000 kit, respectively), with a functional sensitivity below 0.2 ng/mL for thyroglobulin and < 40 IU/mL for Tg-Ab.

### Follow-up

Patients were tested every six months during the first year and then with 6 – 12 month intervals, according to each specialist’s consideration on the basis of every patient’s risk and disease progress.

### Risk re-stratification according to the response to the initial treatment

The response to the initial treatment was established after 2 years of follow-up according to the proposal made by the MSKCC (Memorial Sloan Kettering Cancer Center) (excellent, indeterminate, incomplete response) (12) (Table 2).

**Table 2:** Re-stratification according to the initial treatment by MSKCC (12)

Excellent response	Indeterminate response (any of the following)	Incomplete response (any of the following)
Stimulated and suppressed thyroglobulin < 1 ng/mL	Suppressed thyroglobulin < 1 ng/mL and stimulated thyroglobulin ≥ 1 < 10 ng/mL	Suppressed thyroglobulin ≥ 1 ng/mL
Negative neck ultrasound	Non-specific findings in neck ultrasound or stable subcentimeter nodes	Stimulated thyroglobulin ≥ 10 ng/mL
		Rising Tg values
With no other evidence of disease	Non-specific findings in other imaging studies (CT, NMR, nuclear medicine)	Structural persistence of disease

### Clinical endpoints

Patients were considered disease-free or with no evidence of disease (NED) at the end of the follow-up if they showed suppressed thyroglobulin < 1 ng/mL, non-detectable Tg-Ab, and no functional or structural evidence of the disease. Biochemically persistent disease was considered in those with suppressed thyroglobulin > 1 ng/mL, stimulated thyroglobulin > 2 ng/mL, with no evidence of structural or functional disease; structural persistence was considered in those with evidence of structural or functional disease, and recurrent disease was considered in those with suppressed thyroglobulin > 1 ng/mL and structural or functional evidence of disease after a follow-up period with no evidence of disease. Specific mortality caused by disease and by other causes was also established as the clinical status at the end of the follow-up period.

### Statistical methods

Results are expressed as median and range, and as means ± SD. For statistical analysis, the incomplete and the indeterminate response at 2 years of follow up were gathered into the same group for comparison against the excellent response group. Furthermore, only structural persistence and NED status were considered as clinical outcomes at the end of the follow-up period.

The Fisher Test was used to compare the progress at the end of the follow-up (structural persistence or NED status) among the groups with excellent *vs*. incomplete/indeterminate response within the ATA intermediate risk group.

A p value < 0.05 was considered statistically significant. Analysis was performed using GraphPad Prism version 5, Inc., San Diego CA.

## RESULTS

The characteristics of our 90-patient cohort are described in Table 3.

**Table 3:** Characteristics of the population

		N = 90
Age at diagnosis (years)		
Median (range)	65 (62-82)	
Gender		
Female	86%	77
Male	14%	13
Histology		
Papillary	90%	81
Classic papillary	77%	62
Microcarcinoma	2%	2
Papillary tall cell variant	5%	4
Papillary oncocytic variant	1%	1
Papillary follicular variant	15%	12
Follicular	10%	9
Follicular	67%	6
Follicular oncocytic variant	11%	1
Follicular Hurthle cell variant	22%	2
131I activity for ablation (mCi)		
Mean ± SD	118 ± 41.2	
Median	100 mCi	
AJCC stage		
I	48%	43
II	12%	11
III	27%	24
IVa	12%	11
IVb	0%	0
IVc	1%	1
ATA initial risk classification		
Low	47%	42
Intermediate	43%	39
High	10%	9
Response to therapy (second year)		
Excellent	50%	45
Indeterminate	28%	25
Incomplete	22%	20
Follow-up duration (years)		
Mean ± SD	5.36 ± 4.3	
Median	4	
Range	2-13	
Status at final follow-up		n:90
No evidence of disease	64%	58
Structural persistent/recurrent disease	18%	16
Biochemical persistent/recurrent disease	18%	16
Vital status		n:90
Death of disease	1%	1
Death of other causes	5%	5

Most of the patients were women (86%), median (range) age of the whole group was 65 (62-82) years and papillary thyroid carcinoma was present in 90% of the cases. According to the ATA risk of recurrence categorization, we found 47% of patients in the low risk category, 43% in the intermediate risk category and 10% in the high risk category.

It was observed that, in the low risk category, after the re-stratification according to the response to the initial treatment, 74% showed an excellent response, 24% an indeterminate response and 2% an incomplete response. In the intermediate risk category, 36% showed an excellent response, 51% an indeterminate response and 13% an incomplete response. In the ATA high risk category, 100% of the patients showed an incomplete response.

**Figure 1:**
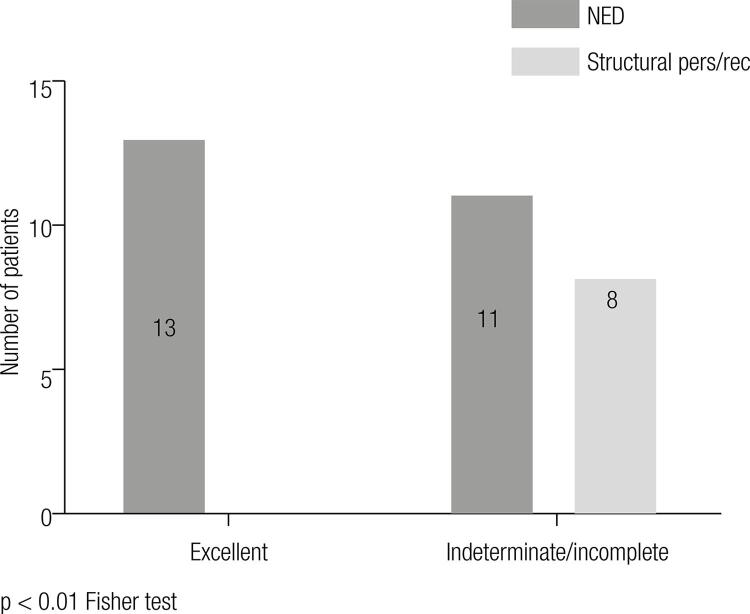
Comparison of the number of patients with no evidence of disease (NED) or with structural persistence or recurrence at the end of the study period between the group with excellent response vs indeterminate/incomplete response to initial treatment within the ATA intermediate risk group.

As regards the clinical status at the end of the follow-up period in the low risk category of recurrence, 95% of the patients were disease-free at the end of the follow-up, in the high risk category 100% showed persistent disease. In the intermediate risk category, 61% was disease-free and 39% showed persistence at the end of the treatment. For this intermediate risk group the re-stratification of the response to the initial treatment proposed by MSKCC showed that those with an excellent response, 100% were disease-free at the end of the follow-up, compared to only 61% in the case of those with an incomplete/indeterminate response, and this was statistically significant among both groups (p < 0.01) (Figure 1). The odds ratio and the confidence interval for disease-free patients at the end of the follow- up between patients with an excellent *vs*. incomplete/indeterminate response within the intermediate ATA category of recurrence was 19.9 (1.035-384).

p < 0.01 Fisher test

Characteristics of the patients that developed structural persistence of disease in the intermediate risk group are shown in Table 4.

**Table 4:** Characteristics of the intermediate risk patients with structural persistent disease

Patient	Age at diagnosis (years)	Sex	Pathological characteristics	I131 (mCi) doses	AJCC stage	Response to initial treatment	Years of follow-up
1	70	F	Papillary with LN mets	150	III	Incomplete	4
2	62	F	Papillary with LN mets	100	III	Indeterminate	4
3	79	F	Papillary with LN mets	150	III	Incomplete	3
4	66	F	Papillary with tall cell	150	IVc	Incomplete	7
5	73	F	Papillary classic variant with LN mets	100	III	Incomplete	6
6	71	M	Papillary classic variant (5 cm) with LN mets	150	III	Incomplete	8
7	66	F	Papillary classic variant with LN mets	150	III	Incomplete	4
8	69	F	Papillary tall cell	100	II	Incomplete	5

LN mets: lymph node metastasis.

Regarding biochemical persistence/recurrence of disease, we found 18% (n = 7) of patients within the ATA intermediate risk category that had an incomplete thyroglobulin response at the end of the follow-up. These patients were excluded from the statistical comparison between patients with structural persistence or free from disease at the end of the follow-up period.

In Table 5 the results of status at final follow up according to the response to therapy are shown.

**Table 5:** Status at final follow up according to the response to therapy

Response to therapy (second year) (n = 90)	NED	Structural persistent disease	Biochemical persistent disease
Excellent (n = 45)	44 (98%)	0 (0%)	1 (2%)
Indeterminate (n = 25)	14 (56%)	3 (12%)	8 (32%)
Incomplete (n = 20)	0 (0%)	13 (65%)	7 (35%)

NED: non evidence of disease.

The specific mortality caused by disease was 1% (n = 1) and the mortality from other causes was 5% (n = 5). The patient with structural persistent disease, who died, was included within the final outcome analysis.

## DISCUSSION

In our study, those patients older than 60 years within the intermediate risk category who, when re-stratified, had an excellent response, did not show an unfavourable impact on the recurrence at the end of the follow-up.

The high or low risk definition is not static and it is possible to reclassify the risk according to the response to the initial treatment. This reclassification or re-stratification validated by MSKCC (12), effectively defines, within the first two years, the short-term risk of having persistent or recurrent disease. Such re-stratification has also been subsequently validated in the study by Castagna and cols. in a shorter period of time (8-12 months) after the initial treatment (13).

As it was expected, in our ATA low risk group of patients, those with an excellent response remained disease-free, without showing recurrent or persistent disease along their progression. Therefore, in the ATA low risk category, the re-stratification confirmed what is already known in the general population with DTC. The same occurred in the ATA high risk category, in which most patients progressed unfavourably and only a minority showed remission of the disease.

According to the literature, the significance of the re-stratification seems to be more relevant in the ATA intermediate risk category, where an excellent response to the initial treatment reduces the risk of recurrence from 18% to 2% (12). In our study, it was precisely in the ATA intermediate risk category where the re-stratification showed its real prognostic value. When patients were reclassified as having an excellent response to treatment within the first 2 years, none showed structural disease at the end of the follow-up, whereas in those with an incomplete or indeterminate response, the ratio of disease-free patients at the end of the follow-up was much lower. These findings would allow for a change in the traditional strategies towards older patients, minimizing the number of studies in the follow-up of many patients who, until now, were controlled and treated with more emphasis.

The high percentage of patients with an indeterminate/incomplete response who, at the end of the follow-up, progressed to a disease-free status can be explained in the studies by Pitoia and cols. (14) and Vaisman and cols. (15). These authors showed that 72% and 34% of patients categorized within the group of indeterminate and biochemical incomplete response respectively, were reclassified as NED without further additional therapy beyond continued levothyroxine suppression (15), similarly, Pitoia and cols. showed that only 16% of patients with biochemical persistence developed structurally identifiable disease over the first 4 years of follow-up. Regarding stimulated thyroglobulin over time, 19% of patients had undetectable values and 65% continued with persistently abnormal stimulated thyroglobulin levels without structural correlate (14).

The idea that advanced age would be a disadvantage in the survival of patients with DTC was proposed by Mazzaferri and Jhiang (16), who showed that the recurrence in the papillary and follicular carcinoma was more frequent at the extremes of life, patients younger than 20 and older than 59 years. For mortality however, rates were increased in patients over 40 years old. In fact, most of the staging systems include age at the diagnosis as a significant prognostic factor (17). In several staging systems, such as TNM, AMES and MACIS, age has been adopted as a factor to be taken into account (18,19). In a study performed by Ito and cols. (20), it was also shown that the prognosis in patients with DTC has a bimodal curve where the risk of nodal recurrence is higher in patients younger than 20 and older than 60 years of age, and that risk of distant metastasis is markedly increased in the latter group.

Although it has been recognized that mortality caused by thyroid cancer would be increased as from 35 years and with the advancing age (21), it has been recently observed that the AJCC staging could overestimate the protective effect of the lower age in patients with DTC, especially in the context of the metastatic disease, resulting in substaging of young patients (22). In fact, young patients with both T3 tumours or with N1b nodal disease have a worse prognosis than older adults within the same TNM stage (17). In addition (23), it has been shown that, in the case of papillary microcarcinoma, the ratio of patients with tumour progression, i.e., progression to clinical disease was lower in adults over 60 than in patients younger than 40 years of age.

Our study supports the theory of the lower impact of age on cancer prognosis since three quarters of this group of elderly patients progressed to disease-free clinical status at the end of the follow-up. However, the most significant contribution of our findings is that, through the restaging after 2 years, according to the initial response to treatment, it is possible to identify the small proportion of patients for whom it is worth to take the utmost care and have a monitoring behaviour.

One of our limitations has been the choice of a determined age cut-off to define older adult. Since this is a study conducted only in 2 endocrine centres, we have not been able to count on a larger population of patients with thyroid cancer in order to study the effects of restaging according to each decade of life. In addition, not having considered the biochemical persistence within the statistical analysis as the clinical status at the end of the follow-up could be another limitation, since according to Miyauchi and cols. (24) the duplication of thyroglobulin within the first two years of follow-up in patients over 60 years of age would be an indicator of tumour growth and distant metastasis.

In our study, we conclude that patients over 60 years of age in the ATA initial low risk category did not show a higher risk of recurrence. On the other hand, in the high risk population, most of the patients progressed to a structural persistence or recurrence at final follow-up. However, the assessment of risk according to the response to the initial treatment after 2 years of follow-up was especially useful in those patients who, at the beginning, were in the category of intermediate risk of recurrence according to ATA. The re-stratification allowed for an early distinction of disease-free patients at the end of the follow-up from those with structural persistence and a worse clinical progress.
